# Reducing bias and enhancing equity in AI-enabled precision nutrition: addressing measurement error across wearables, multiomics, and dietary data

**DOI:** 10.3389/fdgth.2026.1805704

**Published:** 2026-06-04

**Authors:** Andi Mai, Yuanyuan Luan, See Ling Loy, Caihong Qin, Heyang Ji, Ashley Obeng, Mercy Oladuti, Keisuke Ejima, Kai Ting Mok, Satvinder Kaur, Cuiqiong Huo, Joseph Yang, Yang Ou, Omkar Khandpekar, Xiaoxin Yu, Agnes Duah, Velarie Ansu-Baidoo, Aurelian Bidulescu, Marwah Abdalla, Roger S. Zoh, Carmen D. Tekwe

**Affiliations:** 1 Department of Epidemiology and Biostatistics, Indiana University School of Public Health-Bloomington, Bloomington, IN, United States; 2 Department of Reproductive Medicine, KK Women’s and Children’s Hospital, Singapore, Singapore; 3 Obstetrics & Gynecology Academic Clinical Program, Duke-NUS Medical School, Singapore, Singapore; 4 Lee Kong Chian School of Medicine, Nanyang Technological University, Singapore, Singapore; 5 Department of Food Science and Nutrition, Faculty of Applied Sciences, UCSI University, Kuala Lumpur, Malaysia; 6 Department of Psychiatry, Center for Translational Sleep and Circadian Sciences, University of Miami Miller School of Medicine, Miami, FL, United States; 7 Department of Medicine, Columbia University, New York, NY, United States

**Keywords:** algorithmic fairness, artificial intelligence, data integrity, digital health, health equity, machine learning, multiomics integration, statistical calibration

## Abstract

Artificial intelligence (AI) can offer individualized dietary guidance based on multimodal data collected from various sources, including wearable sensors, high-dimensional multiomics and biomarker analyses, behavioral tracking, and self-reported dietary intake, enabling the emergence of precision nutrition. However, the predictive power and fairness of these models rely on the quality of the data inputs, and measurement errors in any of these underlying data streams can introduce systematic bias, degrade model performance, and disproportionately affect underserved populations. In this review, we examine the central role played by measurement error in AI-driven nutrition tools and evaluate statistical and machine learning approaches for mitigating the impacts of measurement error. We provide structured comparisons exploring both classical methods (e.g., regression calibration, Bayesian models) and emerging AI strategies (e.g., denoising autoencoders, multitask learning, uncertainty-aware deep learning) for correcting biased inputs. We also explore how uncorrected measurement error can perpetuate demographic biases, compromise efforts toward personalized medicine, and exacerbate equity gaps when models are deployed in real-world settings. Our review draws upon evidence across nutrition science, digital health, and algorithmic fairness. We propose a framework and offer actionable strategies for overcoming measurement error that can be implemented by researchers, developers, and regulators working at the intersection of data science and dietary health and seeking to build calibration-aware, inclusive precision nutrition systems.

## 1 Introduction

Generalized, one-size-fits-all nutritional guidelines are often inadequate; dietary responses vary widely (1–4), even among individuals (2, 5) with similar demographic or clinical profiles. Personalized nutrition strategies tailored to meet an individual's unique biological and lifestyle characteristics (6–8) can optimize individual health outcomes. In precision nutrition approaches (9–11), chronic disease prevention and management strategies rely on personalized dietary recommendations, determined by integrating individual-level factors, such as molecular (e.g., genetic, metagenomic, or metabolic) markers (12–14), lifestyle choices (15, 16), behaviors (17, 18), and environmental (19, 20) exposures.

Precision medicine has emerged in response to rapid advancements in individualized data collection methods, including high-throughput sequencing and wearable devices that offer real-time monitoring. To process, interpret, and integrate these diverse, large-scale data sets, the precision medicine field has embraced artificial intelligence (AI) (21–26) integration in digital health and nutrition platforms. AI has a remarkable capacity for integrating complex, multimodal datasets, including genomics (27), microbiome analysis (28, 29), metabolomics (30), behavioral data (31, 32), and environmental inputs (20, 33). Existing AI-powered mobile applications (apps) show promise in regulating nutrient intake, aiding weight loss, and managing chronic diseases (15). By seamlessly integrating extensive and heterogeneous data sources (6, 34–38), AI can support clinical decision-making, providing predictive modeling of metabolic responses, risk stratification for chronic diseases, adaptive feedback mechanisms (6), and personalized dietary recommendations (31, 39).

Despite this promise, translating AI-driven insights into actionable recommendations remains difficult. A major barrier is measurement error (40–42), which is inherent to the underlying data sources. For example, self-reported dietary intake data (43–45) frequently suffer from recall biases and underreporting. Data collected by wearable devices or sensors (46, 47) can include errors due to demographic biases (46, 48), calibration drift (46, 49), and inconsistencies across brands (27). Multiomics datasets (e.g., genomics, microbiome data) are susceptible to technical variations and batch effects (50, 51), which can complicate cross-study comparisons and interpretations (27). Left uncorrected, measurement error can introduce systematic biases and variability, undermining model accuracy and reproducibility.

Measurement errors can be classified as either random or systematic, and both types of errors can occur within subjects (i.e., across multiple measures for a single individual) and between subjects (i.e., in comparisons between individuals) (52, 53). Random errors are introduced by random fluctuations in repeatedly collected observational data. Within-subject random errors arise from repeated measurements using the same instrument on the same subject. However, averaging individual replicates can provide unbiased estimates of the true exposure, and random errors tend to diminish in severity with increasing sample sizes (e.g., more measurements taken) over the study duration (52, 53). Even when within-subject random errors are independent of the true exposure and average to zero, these errors can distort the estimated magnitude of associations. Although this noise does not increase the risk of false-positive results (52, 53), the ramifications of these errors are more challenging to predict in more complex models. Within-subject systematic errors consistently deviate in one direction across all individuals and are unaffected by sample size, leading to consistent overestimation or underestimation of effects. Unlike random errors, systematic errors cannot be mitigated by increasing sample sizes when assessing associations. Between-subject random errors reflect individual variability, which can inflate variances without biasing effect estimates. Between-subject systematic errors can be either additive (a constant offset from the true value) or multiplicative (measured values are a fixed multiple of the true values), both of which can result in biased estimates (52, 53). For accurate analyses, all of these errors must be addressed.

Measurement errors can disproportionately impact certain demographic groups (43, 44, 54), exacerbating existing health disparities. For example, populations underrepresented in science may experience greater inaccuracies because the reference datasets used for algorithm training and device calibration may lack individuals with similar characteristics (50, 51). Limited access to high-quality digital tools can further exacerbate disparities, as lower-cost devices may rely on less precise sensors or simplified algorithms, resulting in increased measurement error (55). Moreover, most dietary databases and nutrient estimation algorithms are based on Western diets, but these models may fail to capture nuances associated with cultural variations in dietary patterns, food preparation techniques, and portion-size norms (56, 57). Addressing measurement error in personalized nutrition is both a technical necessity and an ethical imperative for ensuring equitable and effective recommendations for all individuals.

Despite promising results from early studies evaluating the impact of AI-enabled precision nutrition approaches, longitudinal evidence demonstrating durable and reproducible clinical benefits across heterogeneous populations and settings remains limited (6, 58). In addition, many proposed digital nutrition approaches have not yet been evaluated against established, cost-effective, accessible public health or community-based interventions (58, 59), and the real-world impact of digital nutrition tools may be diminished by limited access to expensive wearables or multiomics technologies, leading to differential uptake across populations (60–62). However, this review is not intended to establish the superiority of precision nutrition over standard interventions. Instead, the goal of this review is to examine how measurement error might affect the validity, fairness, and interpretability of AI-enabled precision nutrition systems (40–42, 55, 58) by synthesizing evidence across the fields of nutrition science, digital health, measurement error, and algorithmic fairness. In addition, this review describes common sources of error in AI-enabled precision nutrition; evaluates existing statistical and AI-driven approaches for addressing inaccuracies; and proposes a framework for developing more reliable and equitable systems. We also highlight real-world examples that illustrate how correcting measurement error can improve the reliability, accuracy, and fairness of dietary recommendations. We further provide recommendations for advancing equity and the responsible use of AI in precision nutrition applications to guide researchers, developers, and regulatory bodies. By effectively addressing measurement error, precision nutrition can achieve its full potential in delivering personalized, evidence-based dietary recommendations for improving health outcomes across diverse populations.

## 2 The role and potential of ai in precision nutrition

By addressing several precision nutrition challenges, including dietary assessment, data integration, predictive modeling, and individualized intervention design, AI can deliver scalable, responsive solutions for clinical and public health applications, including real-time intake monitoring and adaptive meal planning. Some AI-enabled precision nutrition systems are designed to only capture and integrate data, whereas others use these data to provide recommendations intended to optimize downstream behavioral or clinical outcomes, such as postprandial glycemic responses (PPGRs), HbA1c values, weight changes, or lipid responses.

When used for dietary assessment, AI can integrate images, audio signals (e.g., chewing or jaw motion), and text descriptions (31, 63–65) to detect foods, estimate nutrients, and predict intake. Machine learning (ML) and deep learning (DL) algorithms, including support vector machines, random forests, and convolutional neural networks, enhance the accuracy of nutrient quantification, particularly when embedded in mobile or wearable technologies (31, 39). For example, ML-driven digital imaging enables real-time, accurate, and convenient dietary intake analysis (32), whereas natural language processing and voice-input systems can enhance user engagement and adherence to dietary tracking (31). AI can also integrate multimodal datasets, constructing comprehensive nutritional profiles for clustering and risk prediction. ML models have been used to stratify patients by nutritional risk using hospital records (66) and predict malnutrition in low-resource settings based on demographic, clinical, and lifestyle data (66, 67). Future systems are expected to support dietary surveillance in large, diverse cohorts through improved intake estimates that recognize complex food characteristics, such as regional variations, cooking methods, and nutrient bioavailability (6, 31, 68), enabling identification of at-risk populations lacking access to specialized nutritional diagnostics.

AI-based predictions of individual metabolic responses to foods enable the development of biologically informed, personalized dietary interventions. Zeevi et al. (28) demonstrated the prediction of PPGRs by an ML model trained using continuous glucose monitoring (CGM), microbiome composition, diet, physical activity, and anthropometric metrics. The Personalised REsponses to DIetary Composition Trial (PREDICT) demonstrated high inter-individual variability in PPGRs and lipid responses using predictive models that incorporated multiomics data and meal context factors (29). Algorithmic models incorporating molecular and behavioral data have also been used to prescribe hypocaloric weight-loss diets (30). These models can deliver tailored feedback when integrated into mobile and web-based platforms used to monitor adherence (69). These systems are evolving into adaptive, closed-loop, decision-support tools that continuously refine recommendations based on updated behavioral inputs, shifting nutrition interventions from static guidelines to real-time, context-aware, personalized dietary advice.

**Figure 1:**
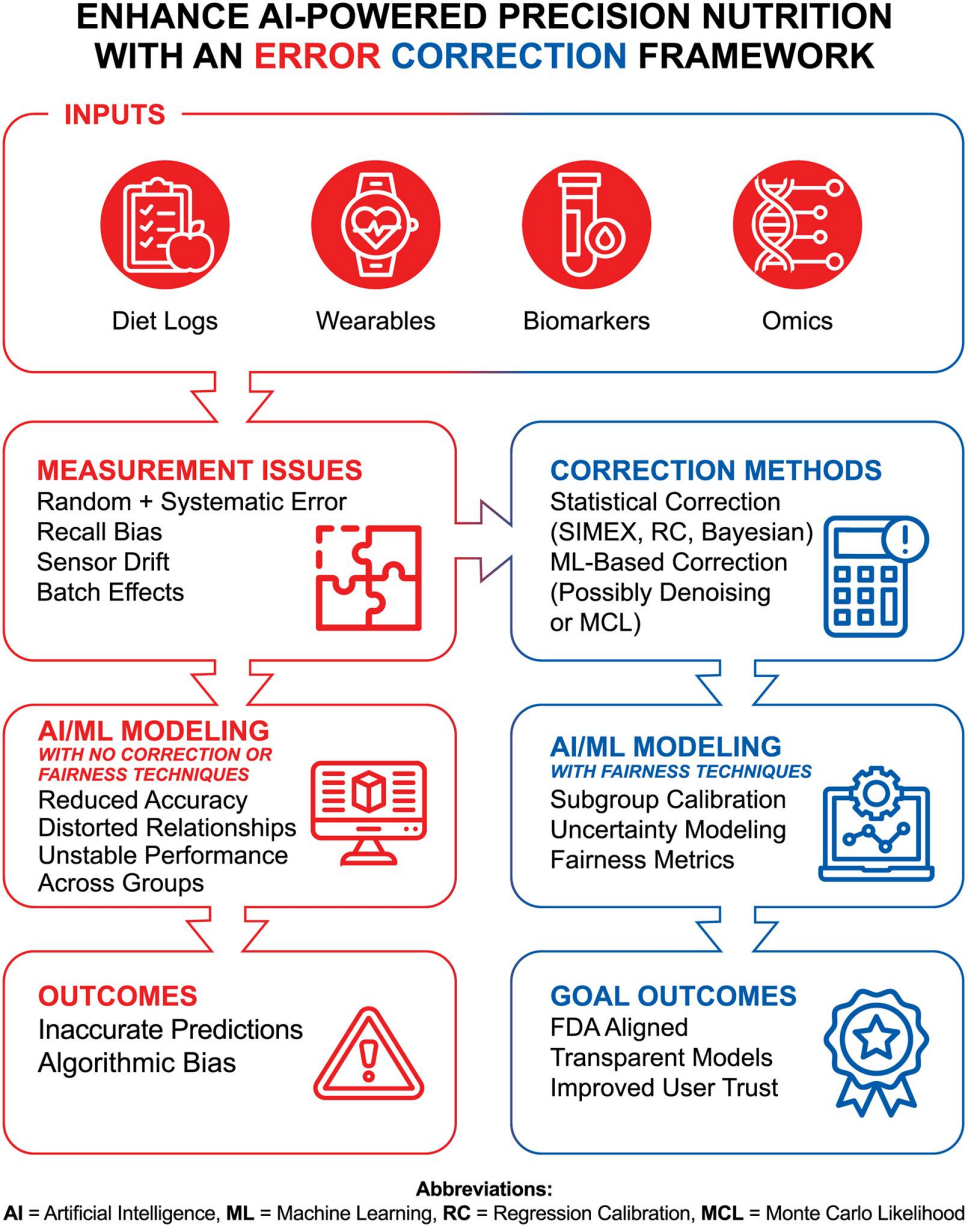
Conceptual figure: integration of data and measurement-error correction in AI-enabled precision nutrition.

The reliability and impact of AI-driven recommendations are fundamentally constrained by the quality of input data, and many core data streams are prone to both random and systematic measurement error. Left unaddressed, these errors can propagate through AI pipelines, distort model training, and obscure true associations. Actualizing the potential of AI to power valid, equitable, trustworthy precision nutrition insights across diverse populations will require rigorous attention to data integrity and detecting, quantifying, and adjusting for measurement error–associated biases. The ideal AI-enabled precision nutrition model (Figure 1) would generate personalized dietary recommendations by integrating multimodal data streams while applying advanced statistical and ML error-correction methodologies that account for common sources of measurement error within each data source.

## 3 Sources of measurement error in precision nutrition

Despite the growing sophistication of AI models, significant sources of measurement error exist at every stage of the precision nutrition data pipeline. Without correction, these errors introduce bias, limit generalizability and equity, compromise performance, and hinder the accuracy of AI-based personalized dietary recommendations. Understanding how these errors originate and propagate is essential for developing robust, equitable AI systems. This section outlines the major sources of measurement error across data modalities commonly used in AI-powered nutrition platforms and highlights their implications for modeling and deployment.

### 3.1 Dietary intake

Accurate assessment of dietary intake is crucial for understanding the relationship between diet and health outcomes. Self-reported dietary intake data, obtained through food frequency questionnaires (FFQs), 24-hour recall, or food diaries, are commonly used in nutrition-focused epidemiological studies (45, 70). However, self-reported dietary intake data are among the most error-prone data sources in precision nutrition; left uncorrected, measurement error in these data (71–74) can introduce bias (75) and profoundly impact analysis, potentially leading to inaccurate conclusions and ineffective recommendations (43, 76). Common sources of measurement error include recall bias, due to individuals misestimating portion sizes or nutritional value, and social desirability bias, in which individuals underreport consumption of foods perceived as unhealthy or overreport consumption of foods perceived as healthy. Systematic misreporting varies by demographic characteristics, including sex, body mass index (BMI), literacy level, and cultural background. Validation studies using double-labeled water and urinary nitrogen consistently show that energy and protein intake are underreported by 20%–30% among individuals with high BMI or low levels of education (43, 44). Supporting this finding, the biomarker validation component of the Observing Protein and Energy Nutrition (OPEN) Study found that energy and protein intake on FFQs were underreported by 35% of men and 23% of women (77). These inaccuracies introduce attenuation bias by weakening estimated associations, reducing signal detection, and impairing inference. Although screening rules [e.g., the Goldberg cut-off, which identifies implausible energy reports based on physiological requirements (78)] are commonly applied to remove extreme reporters before analyses to reduce bias, this approach cannot eliminate bias completely (79, 80); therefore, new approaches are needed to obtain more accurate models.

Dietary intake assessments increasingly incorporate AI-enhanced methods, such as food logs that rely on voice-based tracking or image recognition, to overcome the well-known limitations of self-reported approaches (81, 82). Image-based methods typically use smartphone apps or wearable cameras, together with computer vision and DL algorithms, to capture food intake visually, thereby reducing recall bias and participant burden inherent to conventional self-reported techniques (82). Validity studies reveal that compared with image-based methods, traditional self-reporting is associated with persistent underreporting of energy intake and systematic inaccuracies in nutrient estimations (81, 83). However, image-based dietary assessments remain vulnerable to multiple sources of measurement error, including poor image quality due to inconsistent lighting or food occlusion, visual similarities between different foods, and variability in food presentation (84–86). DL and computer vision systems generally perform best when presented with standardized, well-lit, and separately plated foods, but deviations from these controlled conditions are common in everyday meals (87–89). Errors related to portion-size estimation remain particularly problematic, as volume-to-weight conversions are unreliable, and neither automated algorithms nor human evaluators consistently produce precise results (81).

Furthermore, AI-enhanced methods often struggle with complex meals, regional food types, variations in preparation methods or presentation, and ingredient substitutions (6, 90). Dishes with multiple components, mixed grains, or layered sauces, such as stews, curries, and casseroles, present persistent challenges to segmentation and recognition algorithms, often resulting in portioning and nutrient composition inaccuracies. These challenges become even more pronounced with foods that are underrepresented in the datasets used to train mainstream AI models and speech recognition systems. AI-powered apps frequently fail to identify or accurately estimate the energy content of non-Western and culturally diverse dishes, resulting in significant underestimations of intake [as high as 76% for some Asian mixed dishes (89, 91, 92)], leading to difficulties adjusting for nutrient bioavailability across populations, and limiting the generalizability and accuracy of these methods.

Emerging efforts to address these limitations include the development of more inclusive food image libraries, culturally specific benchmarking datasets, subgroup-specific model evaluation, and community-engaged approaches to dietary data collection and annotation (87, 93–96). However, these efforts remain incomplete, and the broader inclusion of non-Western dietary patterns in training, validation, and deployment pipelines remains necessary to support equitable precision nutrition tools (56, 57, 89, 91, 92, 94, 95).

### 3.2 Biomarkers

Biomarkers, such as glucose, insulin, triglycerides, and inflammatory cytokines, are often treated as gold-standard inputs or outcomes and are used to train AI algorithms that model nutritional status and metabolic responses. However, biomarker levels may fluctuate in response to changes in physiological factors, such as diurnal or circadian rhythms, causing the levels of many metabolic markers to fluctuate significantly depending on the timing of sample collection. Hydration status, fasting adherence, and physical activity preceding the test can further modulate biomarker values. For example, postprandial lipid measures can vary by over 30% depending on timing and recent food intake; therefore, the lack of standardized sampling protocols that account for meal timing is a source of error during model training (97). When these errors are unaccounted for, AI systems may incorrectly learn associations driven more by biological noise than by true exposure–response relationships.

Assay variability, both across and within laboratories, differences in analytical techniques, and sample degradation pose additional risks, especially for multicenter or large-scale studies, leading to batch effects and systematic errors that can undermine model reliability (98, 99). Even minor mislabeling during processing or delayed shipment can introduce significant bias. These factors complicate the use of biomarkers as ground truths or training targets for supervised learning approaches.

### 3.3 Wearable devices

Wearable technologies enable continuous and passive monitoring of physical activity, physiological responses, and metabolic proxies. However, the data generated by wearable devices are susceptible to a range of measurement errors, and without error-correction mechanisms, these inaccuracies may permeate throughout AI models. For example, sensor drift and calibration decay occur over time, especially when users delay firmware updates or wear devices inconsistently (100). Data quality is also affected by user characteristics (age, body composition, gait); activity type and intensity; and variability in device placement, adherence to usage protocols, and synchronization with mobile applications (101–103). For example, wrist-worn devices can miss lower-body movements that may be captured by hip-worn devices (102, 104).

Device heterogeneity is another concern, as different brands and models may produce non-comparable data streams. Because week-long raw signals sampled at high rates are computationally heavy, raw data is often transformed into summaries by proprietary algorithms. Common proxies for intensity include activity counts and monitor-independent movement summary units (105), whereas biologically interpreted summaries include energy expenditure (EE) and heart rate (106). These transformations can introduce modeling error, and device algorithms may differ in their abilities to detect behaviors (107–110). For example, activPAL is more accurate for sitting, standing, and stepping, whereas ActiGraph GT3X+ is best at classifying stepping but is less accurate for other behaviors (111).

Several studies have documented that optical and motion-based sensors, often used in widely marketed wrist-worn devices (e.g., Fitbit), smartwatches (e.g., Apple Watch), or heart rate monitors, demonstrate reduced accuracy for individuals with darker skin tones or higher BMI values (46, 112, 113) due to lower signal-to-noise ratios in optical sensors (114–116). Heart rate errors as high as 16 beats/minute have been reported for people with darker skin tones, with smaller errors (approximately 4 beats/minute) reported for individuals with lighter skin tones (114). Similarly, pulse oximetry has reduced accurate in individuals with darker skin tones, with a positive bias in oxygen saturation readings, particularly at lower true saturation levels (44, 117), which can delay recognition of hypoxemia. This delay can have serious implications for conditions in which timely detection is critical, such as COVID-19. A 2024 study in *Nature Biomedical Engineering* found that AI models built on such device outputs yielded health predictions that were 15%–30% less accurate for Black and Hispanic users than for their White counterparts (46). Such systematic bias may compromise safety monitoring and reduce the effectiveness of prescribed exercise programs.

CGMs are extensively used for glycemic prediction. However, CGMs measure glucose concentrations in interstitial fluid (ISF), and an inherent temporal delay exists for ISF glucose levels relative to blood glucose levels, leading to inconsistent accuracy for CGM values, particularly during rapid glycemic excursions (98–100). The precision of these measurements can be further compromised by interfering substances, calibration inaccuracies, sensor degradation, environmental conditions (106–110), or sensor placement, with discrepancies reported between sensors positioned in close proximity (111, 112).

Ambulatory blood pressure monitors measure blood pressure at regular intervals over a 24-hour period, including during normal daily activities, providing a comprehensive profile of blood pressure variability. Error can be introduced due to improper cuff fit or placement (118), motion artifacts during measurement, or device calibration drift over time (49, 119). Emerging cuffless blood pressure monitoring devices rely on optical, tonometric, or sensor-fusion technologies, which introduce additional challenges related to signal instability, dependence on skin tone and vascular properties, and the need for frequent recalibration against cuff-based measurements. Ambulatory blood pressure monitoring algorithms are often trained on limited demographic datasets, raising concerns about generalizability across populations with varying physiological or anatomical characteristics (120–126). These factors can lead to both random and systematic bias in blood pressure profiles, which are crucial for assessing cardiometabolic risk.

Wearables designed to monitor food intake are gaining in popularity but are also prone to measurement error. Bite counters, wrist-worn sensors that count bites, can misclassify non-eating movements as bites, miss unconventional eating styles (63), and lack context for food types or portion sizes, all of which limit accuracy (127–129). Food intake monitors that rely on cameras worn on the chest or neck gather images of varying quality due to obstructed views, poor lighting, or improper placement or posture (64, 93, 130, 131). Smart glasses with chewing sensors may misclassify eating due to talking or gum chewing, and poor fit, which can lead to inaccuracies (65, 132).

### 3.4 Genome and microbiome data

Precision nutrition models increasingly identify individual susceptibilities and tailor interventions by leveraging high-dimensional multiomics data, including genomics, transcriptomics, metabolomics, and microbiome data. However, the utility of multiomics data is fundamentally constrained by their high dimensionality and biological complexity, which amplify the risk of technical artifacts, measurement error, and reference bias, which can distort AI-driven inference and exacerbate health inequities (133–138). Batch effects during sample processing or sequencing and variability in extraction kits, sequencing platforms, bioinformatics pipelines, and normalization procedures are well-documented systematic error sources that contribute to inconsistencies across studies and obscure true signals (27). Missingness and dropout due to technical failure or sampling burden are particularly problematic in longitudinal designs, resulting in nonrandom data gaps (139). Genomics and microbiome data, which capture host genetic predispositions and gut microbial compositions, respectively, form the biological backbone of many precision nutrition algorithms (13, 140), enabling the modeling of individual metabolic responses (12, 141). However, hybridization failure or low sequencing depth can introduce gaps in genomics studies (142, 143), whereas “false zeros” can be registered in microbiome studies due to low biomass or data dropout, distorting the representation of low-abundance taxa and phylogenetic structures and biasing downstream analyses (144–146). Perhaps most concerning is reference dataset skew: most genomic and microbiome consortia remain dominated by individuals of European ancestry (50, 51), leading to reference databases skewed toward high-income, Caucasian populations. Reference data skew limits the generalizability of AI models to individuals with non-European ancestry and degrades the performance of response predications among individuals underrepresented in research, leading to structural bias in downstream recommendations (147). Failing to correct for these errors risks spurious associations, false discovery, poor reproducibility, and reduced external validity of AI-generated nutritypes or classifiers. Harmonization tools, such as empirical Bayes (148) and the two-step method for removing unwanted variation (149), can mitigate these effects but remain underused in nutrition studies.

### 3.5 Lifestyle and environmental data

AI-driven precision nutrition depends on accurate contextual inputs, such as sleep, stress, physical activity, and medication use. These data are often captured through self-tracking apps, wearable devices, or self-reports, all of which are prone to random and systematic errors. In longitudinal settings, inconsistent logging, device variability, and lack of platform standardization can contribute to missing or inaccurate data, which can distort associations with health outcomes even when other inputs (e.g., physiological signals) are precisely calibrated, ultimately undermining the predictive accuracy and adaptability of AI systems (150–152).

Sleep plays critical roles in energy balance and nutrient metabolism, contributing to chronic disease development (e.g., metabolic disorders) and informing individual dietary behaviors (153). In addition to being associated with increased risks of obesity, type 2 diabetes, hypertension, and cardiovascular disease (154–156), short-duration, disrupted, or poor-quality sleep alters appetite-regulating hormones (e.g., increasing ghrelin and decreasing leptin) (157, 158), extends the eating window, and promotes higher intake of energy-dense foods (159, 160). Furthermore, the relationship between sleep and diet is bidirectional, with unhealthy diets high in saturated fats and sugars impairing sleep quality (161, 162). Integrating sleep metrics into AI models is, therefore, essential for effective dietary personalization.

A core component of precision nutrition is the study of how meal timing interacts with circadian biology to optimize nutrient utilization and overall health (17), also known as chrononutrition. To support metabolic homeostasis, the circadian system favors nutrient intake during the biological day, with fasting at night (18, 163). Evidence from the PREDICT 1 trial showed that the consumption of a meal for lunch elicited a PPGR nearly two-fold that when the same meal was consumed for breakfast (29). These findings reinforce that when food is eaten can be as important as what food was eaten and that consumption timing must be considered in precision nutrition models and recommendations.

Environmental cues, or zeitgebers (time-givers), such as light exposure and physical activity, also influence circadian alignment and eating behaviors (19, 33). Compared to daytime work, night shift work has detrimental effects on metabolic health outcomes, with night shift workers twice as likely to develop poor metabolic health as daytime workers, likely due to circadian rhythm disruption and sleep dysregulation. Exposure to nighttime artificial light can suppress melatonin production, which disrupts sleep and impacts adiposity, contributing to metabolic dysregulation (164). Appropriately timed physical activity can improve circadian alignment, particularly among individuals with cardiometabolic dysfunction (165), whereas exercising at night, when the body is naturally primed for rest, may disrupt circadian phase, alter eating behaviors, and impact downstream metabolic responses (128). These findings highlight the need to consider time-specific exposures or behaviors in precision nutrition frameworks.

Incorporating lifestyle and environmental data into precision nutrition is challenged by several error sources. Lifestyle data on sleep, physical activity, and dietary intake are often collected as self-reported data or by wearable devices (e.g., accelerometers, actigraphy devices). Sleep logs require both subjective and objective input to collect reliable data and capture sleep information throughout the day (including daytime sleep), and a lack of standardized practices for identifying sleep onset often leads to discrepancies between studies (166). Environmental data are often derived from regional sensors or remote databases and may not accurately capture individual-level exposures, particularly in heterogeneous urban or rural settings. An individual's capacity to adopt and sustain personalized nutrition strategies may also be influenced by environmental determinants. For example, neighborhood walkability and access to green spaces may impact the timing and frequency of physical activity (167), whereas food environments, social structures, family routines, cultural norms, and work schedules may impact meal timing and regularity (20). Therefore, environmental determinants must be accounted for when translating AI-driven dietary insights into sustainable, real-world interventions. These methodological challenges highlight the importance of using validated tools, integrating multi-source data, and developing standard protocols to ensure the robust and meaningful application of lifestyle and environmental inputs in precision nutrition frameworks (168).

## 4 Consequences of ignoring measurement error

Measurement error in precision nutrition is both profound and multifaceted, with implications that span statistical integrity, clinical applicability, and health equity. Although AI integration offers unprecedented potential for delivering scalable, personalized dietary interventions, the reliability of AI-powered precision nutrition models depend on the underlying quality of data obtained from diverse modalities, all of which are prone to measurement error (discussed in Section 3, summarized in Table 1). Measurement error that is ignored or inadequately addressed can distort model estimation, compromise individual-level predictions, and introduce systematic bias into deployment pipelines. The technical consequences of uncorrected error include attenuated associations and spurious predictions, whereas the behavioral and ethical ramifications include diminished user trust and unequal performance across populations. Therefore, rigorous error-correction strategies are necessary to ensure reliable and equitable AI-driven dietary recommendations. This section identifies the specific domains for which uncorrected measurement error introduces risk during the development, evaluation, and implementation of AI-powered precision nutrition systems.

**Table 1:** Sources of measurement error in precision nutrition.

Data Type	Source of Error	Implications
Dietary Intake	Recall bias, underreporting, portion misestimation	Attenuation of diet–disease associations
Biomarkers	Diurnal variation, assay error, lab protocol inconsistency	Misclassification of nutritional status
Wearables (CGM, activity)	Sensor inaccuracy, user non-compliance	Inaccurate predictions of metabolic response
Genomics/Microbiome	Batch effects, sequencing error	Spurious associations, poor generalizability
Lifestyle/Environmental	Incomplete or inconsistent tracking	Confounded model predictions

### 4.1 Biased model estimates

Both random and systematic measurement error can distort AI-driven estimates of the relationships among dietary exposures and health outcomes (42, 169). In classical linear regression models, additive error in nutritional variables, such as carbohydrate or energy intake, often leads to attenuation bias: weakened effect sizes make associations appear smaller than they are, masking true associations (42, 170), and larger error variances lead to greater weakening of estimated effects. Although similar attenuation bias can occur in logistic regression, bias is more difficult to quantify in more complex models (171). However, even coefficients of error-free covariates can be biased if the covariates are not independent of the error-prone predictor. In nutritional epidemiology, validation studies reveal that self-reported energy and protein intake (172) are consistently lower than biomarker-derived estimates, implying that diet-related health effects may be underestimated by a factor of two to fourteen (43, 44). Therefore, addressing measurement error is necessary when estimating effects and interpreting diet–disease associations.

Systematic error, including persistent underreporting of specific food groups or nutrients, can result in critical predictors being undervalued or misrepresented, compromising both traditional and ML models. In supervised learning contexts, noisy input features disrupt the learning of meaningful patterns, increase the risk of overfitting, and degrade model generalizability. Recent efforts to improve model robustness, such as the microbiome-based nutrient profile corrector (METRIC), a DL framework designed for microbiome-informed nutritional guidance, incorporate denoising elements to account for variability in input data (173). However, these models continue to struggle when upstream dietary or biomarker inputs are mismeasured. Batch effects in high-throughput multiomics datasets remain persistent sources of bias, often overshadowing biological signals and leading to misleading predictions (27). Collectively, these challenges demonstrate that without rigorous adjustments for biases introduced by measurement error, both classical and AI-based models risk producing inaccurate, non-generalizable outputs that weaken the utility and trustworthiness of precision nutrition tools.

### 4.2 Compromised personalization accuracy

The PREDICT 1 study revealed substantial inter-individual variability in PPGRs and lipid responses, highlighting the need for highly personalized models (29). However, predictive models are vulnerable to performance degradation if measurement error in the underlying data is not appropriately accounted for, threatening the accuracy of individualized recommendations for dietary intake, nutrient goals, meal compositions, or meal timing (60, 174). For example, the presence of motion artifacts in CGM data markedly reduced model prediction accuracy, even when using highly optimized ML models (175). The MealMeter model, which estimates macronutrient intake by integrating computer vision and metabolic signals, demonstrated high accuracy under laboratory conditions, but inconsistent logging and wearable signal degradation in free-living environments led to marked accuracy reductions (176). Recent evaluations of nutrition apps revealed systematic overestimation and underestimation of energy intake for both manual logging and AI-enabled food image–recognition platforms, and errors varied according to dietary patterns (e.g., Western vs. Asian diets), with reduced accuracy for culturally diverse foods (90). Although advances, such as RGB-D (Red, Green, Blue channels combined with depth) multimodal frameworks with ingredient guidance, show potential to improve prediction accuracy for complex meals, these models continue to face limitations (background clutter, mixed dishes, occluded portions, and variability in lighting or presentation) in real-world deployment (177). Therefore, precision nutrition models must consider and adjust for sources of measurement error in real-world settings to ensure the delivery of accurate and effective recommendations.

### 4.3 Reduced user trust and engagement

User engagement with digital nutrition platforms is strongly influenced by the perceived accuracy and relevance of AI-generated feedback. Although a 2024 scoping review of AI-assisted dietary tools (e.g., image-based logging, portion detection) found that accuracies ranged from 74% to nearly 99% for food recognition and nutrient estimation (31), users often cease engagement when feedback does not reflect their lived dietary experiences or physiological sensations; mismatches between model outputs and physiological states, such as an app reporting sufficient caloric intake when users feel fatigued, undernourished, or hungry (31, 32), can rapidly erode trust (178). This issue is compounded by the lack of transparency around data uncertainty. For example, some apps present glucose or risk predictions as precise numbers or simple traffic-light labels, without indicating that readings may be noisy, incomplete, or reflect model-based estimates. People with limited digital or health literacy may place too much confidence in the displayed values, experience confusion when the readings do not match how they feel, or develop distrust when they receive conflicting advice or disparate readings from different apps or between apps and clinicians (179, 180).

Furthermore, AI models trained on noisy or biased data can learn spurious associations. For example, batch effects in microbiome datasets can cause models to erroneously cluster individuals by laboratory processing site rather than underlying biological patterns (27). These artifacts may lead to misclassification of nutritional risk or overreliance on unstable features, reducing model interpretability and generalizability. If used in clinical or consumer-facing systems, such flawed predictions can result in ineffective or misleading guidance, raising both safety and ethical concerns (58, 181). Therefore, the failure to address measurement error and data integrity can negatively impact model performance, with potentially damaging downstream effects on long-term user engagement and public confidence in digital nutrition technologies.

## 5 The need for precision measurement error–correction methods

Measurement-error correction upholds scientific integrity, supports compliance with regulatory and funding requirements, improves model performance and interpretability, and promotes equity and user trust. As AI-driven approaches become embedded in nutrition science, healthcare delivery, and consumer-facing platforms, the application of rigorous, context-sensitive, measurement error–correction approaches will ensure the development of scalable, adaptive digital nutrition systems that function reliably across diverse populations and real-world settings. This section presents both policy-driven and application-focused justifications for embedding measurement error–correction approaches within the core architecture of these systems and throughout the full data-processing pipeline, including data collection, preprocessing, model training, and deployment.

### 5.1 Policy-driven justifications

#### 5.1.1 Regulatory compliance

Global regulatory and ethical frameworks increasingly emphasize that AI-enabled systems must demonstrate transparency, robustness, and equitable performance across diverse populations. Because uncorrected error can obscure subgroup variability, inflate apparent accuracy, and hinder regulatory approval (182), the U.S. Food and Drug Administration (FDA), the European Medicines Agency (EMA), the American Medical Association (AMA), and the World Health Organization (WHO) require evidence that algorithms perform consistently across demographic groups and that potential measurement error is explicitly addressed (59, 183, 184). The FDA's 2025 draft guidance for AI-enabled medical devices explicitly requires sponsors to describe how measurement error or uncertainty is assessed and mitigated, underscoring that correction is now considered a prerequisite for approval and responsible deployment (185). Ethical guidance from WHO and the AMA further stresses the need to reduce bias, involve affected communities in evaluation, and clearly communicate the limits of data and predictions (59, 184). To comply with regulatory expectations and ethical standards, pipelines are encouraged to document input data quality and sources, incorporate fairness-aware training, integrate subgroup calibration, validate outcomes across demographically representative cohorts, and report uncertainty in accessible terms.

#### 5.1.3 Funding and scientific rigor

Major funders, such as the National Institutes of Health (NIH), Wellcome Trust, and Horizon Europe, increasingly prioritize reproducibility, demographic representativeness, and data integrity. For example, recent NIH R01 and U01 funding opportunities in precision nutrition explicitly requested proposals addressing measurement error in sensor-collected and self-reported data (186). In parallel, leading peer-reviewed journals are raising expectations for model transparency and data quality, with systematic reviews now flagging studies that omit measurement error–correction strategies as methodologically limited.

### 5.2 Application-Focused justifications

#### 5.2.1 Improved model accuracy and interpretability

AI models trained on uncorrected dietary, sensor, and multiomics data are vulnerable to attenuation bias, spurious feature selection, and unstable effect estimates, particularly in high-dimensional and longitudinal settings. Empirical studies demonstrate that measurement-error correction yields substantive gains in model calibration, inferential stability, and interpretability, with associated improvements in predictive performance under realistic error structures.

Simulation and validation-based analyses of nutrition and biomarker studies reveal that measurement error–correction models using regression calibration (RC) or Bayesian hierarchical approaches can substantially improve the accuracy of exposure–outcome associations, reducing attenuation bias by 20%–60% depending on measurement reliability, error structure, and study design (42, 170). In applied analyses, these approaches lead to meaningful changes in effect estimates and inferential conclusions, reducing attenuation bias, improving uncertainty calibration, and enhancing alignment with biological plausibility, though the degree of improvement achievable varies across datasets (187). Bayesian models that jointly represent latent true intake and observed noisy measurements can yield more stable parameter estimates and covariate importance patterns across repeated samples, enhancing the interpretability and reproducibility of dietary risk factor assessments (42, 171, 187).

Simulation studies introducing controlled measurement error in digital health and microbiome-enabled nutrition models show that denoising autoencoders (DAEs) and probabilistic calibration layers that explicitly account for measurement error and data uncertainty improve estimation accuracy, reduce bias, and enhance model stability relative to models trained on uncorrected inputs, including substantial reductions in mean squared error and improved robustness of learned representations (188, 189). For example, the METRIC framework integrates probabilistic calibration to address microbiome measurement noise, resulting in better calibrated and more robust models that predict PPGRs and lipid responses with more biologically coherent feature attributions (173). Complementary evidence from simulation and applied nutrition studies of self-reported dietary data demonstrates that compared with naïve, uncorrected models, models that use mixed-effects or SIMEX-based approaches to explicitly correct for measurement error improve inferential validity, yielding substantial reductions in estimation bias, more reliable uncertainty quantification, and improved recovery of heterogeneous exposure–outcome relationships (54, 190). These findings demonstrate that beyond being a theoretical safeguard, measurement-error correction serves as a practical mechanism for enhancing performance by improving estimation accuracy, stabilizing inference, and better aligning model outputs with underlying biological mechanisms, which may incentivize AI developers to incorporate these methods into precision nutrition pipelines.

#### 5.2.2 Enhanced user trust and feedback systems

User trust is integral to the adoption of and sustained engagement with digital nutrition tools (188). When models trained on noisy dietary, sensor, or biomarker data present point estimates or definitive recommendations without communicating uncertainty, users may be misled, particularly when recommendations fall within the margin of measurement error, eroding trust and engagement (189, 191). AI developers can improve user-facing design by integrating measurement error–correction approaches that offer feedback systems for quantifying and transparently communicating uncertainty related to data quality, device reliability, and confidence in predicted responses, rather than presenting recommendations as uniformly certain. For example, incorporating sensor calibration metadata and accounting for device-level error into CGM data analysis tools can reduce overconfident and inconsistent recommendations, improving glycemic management and sustaining user trust (29). Platforms that explicitly display uncertainty measures, in the form of reliability scores, confidence intervals around predicted responses, or adaptive recommendations that downweight low-quality data, enable users to make better-informed decisions, which improve both adherence and satisfaction (68).

#### 5.2.3 Cross-population generalizability

Correcting for subgroup-specific errors through stratified calibration, such as adjusting CGM predictions according to skin tone, body composition, or cultural dietary patterns, can significantly reduce error variance and enhance both fairness and performance. A 2024 study demonstrated that explicitly modeling optical sensor calibration bias improves predictive accuracy by over 25% in African American and Latino cohorts (69). These adjustments move beyond performance optimization, serving as practical mechanisms for reducing health disparities and advancing equity in digital health.

#### 5.2.4 Data lifecycle optimization and real-time correction

Advanced AI systems now support continuous model refinement through real-time reweighting of noisy data streams at multiple points in the digital health pipeline, including device-level sensing, application-level data aggregation, and downstream data integration and modeling, preventing overreaction to transient measurement artifacts. In practice, this approach allows digital health platforms to adjust the influence of incoming sensor data based on reliability signals. For example, platforms may downweight accelerometer data during non-wear periods or CGM values during periods of calibration uncertainty, resulting in more stable and trustworthy user-facing recommendations. Multitask learning (MTL), hybrid calibration–ML architectures, self-supervised error detection, and other techniques enable adaptive correction at the data integration and modeling layers by explicitly modeling shared structures across related physiological signals, incorporating device-level calibration information into prediction pipelines, and automatically identifying and downweighting corrupted or low-reliability data segments during training and deployment (31). Importantly, correction-aware systems explicitly track data provenance and uncertainty to accommodate partially or pre-corrected inputs to avoid overcorrection and limit error compounding and propagation across the computational pipeline. These approaches are particularly relevant for long-term use cases, such as chronic disease management, for which uncorrected errors across multiple sources can lead to fluctuating data quality. Embedding measurement-error correction as a core system function at the integration and modeling layers enables continuous learning and ensures durability, accuracy, and scalability of AI-enabled nutrition platforms.

## 6 Statistical and AI-based solutions for measurement error

Both classical statistical approaches and AI-based methods have been developed (Table 2) to address measurement error, resulting in a growing toolbox available to improve data integrity, strengthen model accuracy, and enhance equity when developing AI-enabled nutrition systems. This section provides an overview of these methods, focusing on the practical deployment, underlying assumptions, strengths, limitations, and implications of each method for individual dietary recommendations and population-level inference.

**Table 2:** Toolbox for measurement error–correction methods in precision nutrition.

Method	Approach	Strengths
Regression Calibration	Use of validation data to correct exposure bias	Widely used, interpretable
Instrumental Variables	External proxies for unmeasured truth	Can address endogeneity
Denoising Autoencoders	Deep learning reconstruction of clean signal	Good for high-dimensional inputs
Bayesian Models	Model uncertainty and error explicitly	Flexible, accommodates prior knowledge
Hybrid Calibration + Machine Learning	Combine statistical and deep learning methods	Balances robustness and adaptability

### 6.1 Classical statistical methods

#### 6.1.1 Regression calibration (RC)

One of the first methods developed to address measurement error in health research (192, 193), RC [detailed in (41)] remains widely used in regression models that link exposures to health outcomes due to its simplicity, practicality, and efficacy. The central concept of RC is the replacement of error-prone measures using more accurate approximations derived from available validation or replicate data. This approach has been extensively applied in nutritional epidemiology to address measurement error (44, 117, 170, 194–197), such as using biomarker-based values (e.g., double-labeled water or urinary nitrogen) to correct for error in self-reported dietary intake data (44, 170). These corrected exposures can then be incorporated into models, including AI pipelines, linking diet to health outcomes. The use of calibrated dietary inputs can reduce attenuation bias, improve model generalizability, and enhance predictive stability. Recent RC extensions are more flexible, including applications for longitudinal data that may contain errors that are correlated over time (198). When supported by appropriate validation data, RC can meaningfully reduce bias, improve statistical power, and strengthen the credibility of findings in population health studies.

#### 6.1.2 Instrumental Variable (IV)

When reliable validation data are not available, IV methods can be used to address both systematic error and unmeasured confounding. Particularly useful in dietary studies, IV methods use an auxiliary measure (referred to as an instrument) that is related to the true exposure but not directly linked to the outcome (42). For example, biomarker data that correlate with dietary intake but are not directly associated with the health outcome (except through effects on dietary exposure) can serve as the instrument for dietary intake data derived from FFQs or 24-hour recalls (199). Genetic variants (e.g., Mendelian randomization) and device-derived signals (e.g., passive wearable data) can also serve as valid instruments for obtaining unbiased dietary effect estimates (200). Recent work has strengthened IV approaches by using multiple or weaker instruments and by incorporating ML techniques to improve performance (201–204). Although originally developed for linear models (205, 206), IV methods have been extended to many types of regression, including those used in survival and longitudinal analyses (201–204, 207–210), and can be used to address error in both scalar and functional covariates (211–214). IV methods have become important tools for addressing systematic error and bias in nutritional epidemiology research, making study findings more reliable and better able to inform health recommendations.

#### 6.1.3 Validation and replicate Sub-studies

Validation and replicate studies provide empirical data that can be used to estimate measurement-error variance. In these studies, the same individuals complete repeated assessments over a defined period, which allows partitioning of within-person and between-person variability. Replicates can be obtained from repeated 24-hour recalls, multi-day weighed food records, or device-based measures, such as CGM or step counts. Such designs support classical error modeling and are vital for establishing the reliability of exposure measures (42). By treating the true value for each subject as fixed across sessions, measurement-error variance can be estimated across sessions. Replicate data can also be used to obtain approximations of true measures in a mixed-effect model setting under a classical error model (215–217) by modeling the observed measure as a function of the true measure and a random error term (215).

#### 6.1.4 Bayesian hierarchical models

Applications in nutritional epidemiology have shown that Bayesian hierarchical models can reduce bias and recover true associations by explicitly accounting for systematic reporting errors and heteroscedastic variability (216–218). Unlike standard RC methods, which rely on linearity assumptions and the conditional expectation of the true exposure given its surrogate, Bayesian hierarchical models jointly model the true exposure, error-prone surrogates, and the health outcome, providing a flexible framework for correcting bias in complex, nonlinear settings or when validation data are sparse. A major strength of Bayesian modeling is the ability to integrate multiple sources of information and flexible error structures within a single model, allowing joint analyses and sharing of information to account for measurement error, batch effects, and differences between individuals through hierarchical latent structures (219, 220). Unobserved true exposures are represented as latent variables, and the hierarchical model jointly specifies [1] their distribution in the population, [2] the measurement models for different instruments (such as FFQs, 24-hour recalls, or biomarkers), and [3] the relationship between the true exposure and the health outcome of interest. Hierarchical frameworks can accommodate various measurement error sources, incorporate prior knowledge, and combine complementary measures, such as recalls and biomarkers, to produce calibrated exposure estimates (215, 221, 222). In simulation studies, Bayesian approaches are particularly useful when exposure–response relationships are nonlinear, models must account for error-prone surrogates, or substantial between-subject heterogeneity must be addressed, yielding less biased effect estimates than RC- or IV-based approaches, which are inadequate for more complex situations.

Seminal work by Sinha et al. (219) introduced a semiparametric Bayesian framework that enabled the simultaneous correction for random and systematic self-reported bias while accounting for individual heterogeneity in dietary distributions. Bayesian approaches have also been extended beyond dietary intake to include biomarker and multiomics settings. Pittavino et al. (220) proposed a multilevel, latent-factor, Bayesian model that accommodates both within- and between-individual variability. Reynolds et al. (223) developed a fully Bayesian model that includes biological covariates, harmonizes features, and supplies calibrated posterior uncertainty that can be propagated to downstream analysis. Similarly, Ohn, Lin, and Kim (224) developed a Bayesian sparse factor model that captures differences between individuals while borrowing strength across the cohort. One appealing advantage of Bayesian approaches is the ability to fully recover the distribution of latent covariates. Bayesian deconvolution has been a very active research area [see Sarkar et al. (225)], and Bayesian hierarchical measurement–error models have recently been extended to functional and wearable-derived exposures. Zoh et al. (2024) developed a Bayesian semiparametric scalar-on-function regression framework that corrects for measurement error in function-valued covariates, such as wearable-derived physical activity or EE trajectories, using both IVs and flexible priors (213). When applied to accelerometry data from a large population-based study, the model yielded less attenuated exposure–outcome associations than naïve models while generating calibrated posterior uncertainty parameters suitable for downstream analyses.

### 6.2 ML and AI-based strategies

#### 6.2.1 Denoising autoencoders (DAEs)

DAEs are DL models designed to reconstruct clean signals from inputs corrupted by noise, effectively capturing robust data representations (226, 227). By forcing networks to recover the original data from deliberately noised inputs, DAEs naturally filter out irrelevant variability, enhancing data quality and reducing measurement error. DAEs have improved the accuracy of patient-reported carbohydrate data by leveraging higher-quality CGM data (228), and multimodal stacked DAEs have been used to impute missing values in large-scale dietary surveys, achieving higher accuracy and computational efficiency than traditional imputation methods (229). Furthermore, DAEs have increased the robustness of near-infrared spectroscopy data to random noise, significantly improving predictive modeling for nutrient quantification (230). Despite these advantages, DAEs face practical limitations, including the risk of overfitting with limited training samples, reliance on adequate noise modeling, and challenges in interpreting latent representations (231, 232). Multitask Learning (MTL).

MTL is an ML paradigm that simultaneously leverages related prediction tasks, allowing models to learn shared representations that enhance robustness and predictive accuracy across multiple outcomes (233, 234). By jointly modeling correlated nutritional and clinical data streams, MTL can reduce noise and improve inference reliability. MTL models that integrate dietary images, meal descriptions, and user-specific data outperform single-task methods in automated dietary assessment, enabling more accurate dietary feedback (235, 236). Similarly, an MTL approach incorporating genomics, nutritional data, and clinical indicators improved metabolic syndrome prediction by modeling multiple components simultaneously, yielding superior performance over traditional single-task methods (237). Despite its promise, MTL faces challenges, such as negative transfer between unrelated tasks, increased computational complexity, and sensitivity to task selection, and effective management of these issues requires careful task grouping and regularization strategies (238).

#### 6.2.2 Ensemble learning

Ensemble learning methods integrate predictions from multiple base models to improve robustness, accuracy, and generalization (239–241). By aggregating diverse algorithms, such as random forests and gradient boosting, ensemble techniques capitalize on the complementary strengths of individual models to reduce variance and bias in complex, noisy data settings (242, 243). Ensemble random forest models resulted in higher dietary recommendation accuracy than neural networks (244), and hybrid ensemble approaches significantly enhanced malnutrition prediction among children (245) and achieved high accuracy in obesity classification based on lifestyle and self-reported behavioral data (246, 247). However, ensemble models can be computationally intensive, require careful hyperparameter tuning, and may lack transparency, highlighting the need for rigorous validation and interpretability strategies (240, 241).

#### 6.2.3 Uncertainty-Aware deep learning (DL)

Uncertainty-aware DL encompasses neural network frameworks that quantify prediction uncertainty, providing valuable insights into model reliability and supporting safer decision-making in clinical and nutritional contexts (248, 249). By incorporating probabilistic reasoning, methods such as Bayesian neural networks (250) and Monte Carlo dropout can estimate prediction confidence and distinguish between model-based and data-based uncertainty (251). These capabilities are particularly important in personalized nutrition, where low-confidence predictions, such as uncertain glycemic responses, might prompt caution or user intervention. In food nutrient estimation, uncertainty-aware models using mobile near-infrared spectroscopy devices achieved high accuracy, automatically rejecting unreliable predictions and improving safety for vulnerable groups (252). Similarly, uncertainty quantification enhanced reliability in health monitoring systems with multi-source noisy data, improving decision support in critical environments (253). However, Bayesian inference, Monte Carlo sampling, and similar techniques are computationally intensive and often require large, labeled datasets, limiting scalability. In addition, uncertainty estimates can be difficult to interpret and communicate to end-users, particularly in clinical settings (248). Addressing these issues is critical for the broader adoption of uncertainty-aware DL in precision nutrition.

#### 6.2.4 Semi-supervised learning and self-training

Semi-supervised learning and self-training are ML paradigms designed to leverage abundant unlabeled data alongside limited labeled examples to improve model accuracy, scalability, and robustness (254–256). These approaches are especially valuable in settings where labeled data are expensive or time-consuming to obtain, as in nutritional studies involving biomarkers or dietary assessments. An initial model, trained using labeled data, iteratively assigns pseudo-labels to unlabeled examples based on prediction confidence, and then the model is retrained to refine performance (254, 256). By reducing the number of required labeled samples, semi-supervised learning has significantly improved data efficiency in spectroscopy-based food safety assessments (257), and self-training methods have successfully been used to adapt dietary behavior recognition models to individual eating patterns, enhancing personalized meal intake analysis (258). However, incorrect pseudo-labels can propagate errors, and iterative retraining cycles are associated with increased computational costs; therefore, robust initial labeled datasets and careful thresholding strategies are needed to balance data quantity and quality (254, 256).

#### 6.2.5 Hybrid statistical–AI models

Hybrid statistical–AI models integrate classical statistical methods with modern AI techniques, combining the interpretability and domain knowledge of traditional models with the scalability and predictive power of ML. These models are particularly valuable when measurement error and data sparsity are common. For example, RC-adjusted intake data or Bayesian priors from classical models can be embedded into neural architectures to improve inference in small or noisy datasets. In low-resource settings, a hybrid model combining clustering with extreme gradient boosting fuzzy classification achieved high accuracy in classifying childhood nutritional status, enabling early malnutrition detection (259). Similarly, a systematic comparison using the National Health and Nutrition Examination Survey (NHANES) dataset showed that hybrid models substantially outperformed traditional techniques in identifying hypertension risk, demonstrating superior handling of imbalanced nutritional and lifestyle data (260). Moreover, hybrid systems integrating natural language processing with ML generated higher-quality, personalized dietary recommendations from large-scale dietary datasets than single-method approaches (261). However, hybrid models are associated with increased computational complexity, require careful integration to preserve interpretability, and rely on expert-driven design choices. Rigorous validation and thoughtful model construction are needed to ensure real-world applicability in precision nutrition.

### 6.3 Integration across multimodal systems in precision nutrition

Advancing precision nutrition relies fundamentally on the integration of multimodal data, each characterized by distinct sources of measurement error and biological variability. Effective correction strategies must be tailored to the statistical and biological properties of each modality (262).

Self-reported instruments used to monitor dietary intake, such as FFQs and 24-hour recalls, are prone to recall bias, underreporting, and portion-size misestimation. Statistical models that account for systematic reporting error can be combined with AI-driven tools, such as image-based food recognition systems and representation learning approaches, to enhance the accurate identification of complex meals, culturally specific foods, and varied preparation methods (263). Nutritional biomarkers, particularly metabolites, can be combined with self-reported instruments, offering functional insights into physiological and nutritional status and serving as key inputs for AI-driven predictive models informing individualized dietary recommendations (264, 265). However, high-dimensional molecular biomarker data are prone to batch effects, assay drift, and platform-related variability. Preprocessing strategies, such as Bayesian latent-factor models and DL architectures with batch normalization and artifact-detection components, can support harmonization across datasets (264). In addition, wearable biosensors have been developed to conduct non-invasive, real-time tracking of nutrient intake and metabolic biomarkers (266, 267). However, wearable devices, including CGMs, accelerometers, and smartwatches, face challenges that include calibration drift, signal dropout, and demographic bias (e.g., reduced performance for darker skin tones or atypical user motion). Correction strategies for these data may incorporate device metadata, environmental context, and user adherence profiles to enable real-time signal calibration (266, 267). By combining multiple error-corrected data modalities, AI-based models can provide the most accurate and personalized nutritional recommendations to individuals.

### 6.4 Deployment considerations and ethical implications

AI models must output interpretable predictions, accompanied by reliability scores or uncertainty estimates, particularly in clinical or user-facing environments (268, 269), to help end-users understand the confidence associated with dietary recommendations (270). A well-calibrated model yields predictions that match observed outcomes, both overall and within relevant subgroups. Tools, such as reliability diagrams, expected calibration error metrics, and clear uncertainty reporting, are essential for evaluating model performance and ensuring that predictions are accurate, interpretable, and suitable for real-world decision-making (271–274). Models would ideally be assessed using these tools prior to deployment.

To support long-term engagement, AI systems are encouraged to incorporate real-time correction mechanisms to enhance recommendation accuracy and foster trust. For example, CGM predictions can be adjusted based on time-of-day variability, device-specific reliability scores, or user confirmation of meal data (275, 276). To avoid substantial variation, both within sensors over time and across different sensors worn simultaneously, data inputs are recommended to be validated across devices and parameters (CGM, wearables), populations (age, sex, socioeconomic status, race, ethnicity), and settings (free-living vs. controlled) (277–279).

Personalized, AI-based nutrition tools will only be adopted if they are viewed as useful and easy to use (61, 211). Cultural, racial, and ethnic factors may also influence data interpretation and user expectations, underscoring the need for proactive, equitable design (62). Therefore, in addition to accuracy, prediction models are encouraged to be developed with consideration for fairness, equity, and cultural sensitivity, with model refinement guided by fairness metrics that acknowledge and estimate group-specific error structures (e.g., bias, variance, and misclassification rates), such as equalized odds or subgroup calibration. Measurement error often varies across population subsets defined by race and ethnicity, sex, age, primary language, BMI, device type, or clinical setting. As a result, even with error correction, a model may be less accurate for Black adult users than for White adult users and require adjustment to ensure equitable performance across groups (270). To prevent algorithmic bias and ensure that models are appropriate for all users, validation and correction strategies are encouraged to be developed for population subsets that match real users.

## 7 Potential real-world applications of AI-based precision nutrition models

Conventional mechanisms for capturing dietary exposure are known to introduce nontrivial measurement error; however, dietary studies do not always explicitly implement approaches for addressing such error, even though uncorrected error can attenuate model performance and bias individualized recommendations. By contrast, studies involving wearable sensors offer several well-documented examples in which measurement-error correction substantially improves measurement accuracy under free-living conditions, highlighting that such correction is essential for the delivery of accurate, equitable dietary personalization based on wearable sensor data. An integrated, AI-based, precision nutrition model will likely need to address measurement error across many different inputs, including self-reported data, biological marker data, and wearable sensor data. The following examples describe potential applications for ML- and DL-based precision nutrition models, demonstrating their potential to guide clinically meaningful improvements in glycemic control and weight outcomes, even when they treat key inputs (diet, CGM, microbiome) as error-prone but essentially observed. These examples were selected to illustrate how correcting measurement error in dietary, wearable, and related behavioral data can strengthen downstream inference and personalization and are not intended to provide a comprehensive review of specific longitudinal exposure–outcome relationships. These examples also emphasize that unleashing the full potential of AI-based precision nutrition will likely require that explicit error-correction strategies be embedded into model pipelines to materially improve predictive accuracy, personalization, and model performance. In addition, we provide recommendations for how future AI-based precision nutrition models can build on and extend current approaches.

### 7.1 Example 1: multiomics prediction of postprandial glycemic responses (PPGRs)

In a large cohort of roughly 800 healthy adults (28), an ML model was able to combine week-long CGM data with information on clinical blood markers, gut microbiome composition, habitual diet, anthropometrics, and physical activity to accurately predict individual PPGRs to meals in a real-world, uncontrolled setting. In a blinded, randomized dietary intervention, individuals were assigned to receive either control diets based on standard dietary advice or model-selected meals predicted to produce smaller PPGRs; the model-selected meals consistently yielded lower PPGRs and more consistent shifts in gut microbiota than the control meals. Although this approach did not explicitly incorporate formal measurement error–correction methods for the self-reported or inferred dietary and lifestyle inputs, it illustrates how residual error in food logs, microbiome profiling, and CGM could propagate through multiomics models and subtly bias individualized meal recommendations. From a design perspective, future AI-based precision nutrition models that build on this work are encouraged to embed explicit calibration or measurement-error models for key inputs to prevent predictive gains from multiomics integration from being undermined by systematic misclassification or underreporting in any single data stream.

### 7.2 Example 2: glucose-guided eating via a data-driven fasting app

A data-driven fasting app was developed to operationalize a “glucose-guided eating” algorithm, which encourages individuals to delay or initiate meals based on CGM readings and logged food intake. In a large, real-world observational analysis of the first 30 days of app use among more than 6,000 adults, users showed consistent weight loss across baseline BMI categories and small but clinically relevant improvements in fasting glucose, with the largest average reductions observed among individuals with baseline prediabetes or diabetes (280). In this implementation, the algorithm effectively treated CGM readings and self-logged mealtimes as observed, without explicit modeling of sensor noise, calibration drift, or logging inaccuracies, even though these are known error sources in free-living settings. Incorporating explicit error correction will be critical for future AI-based precision nutrition systems that adopt similar “when to eat” feedback, ensuring safe and reliable guidance as algorithms are scaled to more diverse devices, eating patterns, and user populations (280).

### 7.3 Example 3: digital twin–enabled precision nutrition for type 2 diabetes

A digital twin platform was developed to integrate CGM data, detailed dietary logs, physical activity data, medication information, and other biometric inputs into individualized ML models for people with type 2 diabetes. These digital twins were used to generate real-time predictions of PPGRs to candidate meals and lifestyle choices, and early real-world evaluations of the program reported substantial reductions in HbA1c, weight, and insulin resistance indices, with some participants achieving diabetes remission. The digital twin models relied on high-frequency CGM and self-reported dietary data but did not describe measurement error–correction procedures for these inputs, despite well-recognized measurement error sources for both data types. Explicit modeling of uncertainty and measurement error across heterogeneous data streams, including diet, CGM, medications, and physical activity, will be essential for next-generation AI-based precision nutrition models that extend this paradigm, ensuring that digital twin predictions remain accurate, transportable, and equitable when deployed beyond tightly monitored program settings (281).

### 7.4 Example 4: weight management and obesity treatment

AI-based precision nutrition models are increasingly employed to enhance weight management by predicting which dietary or supplement strategies are likely to be most effective for an individual rather than relying on a single, standard plan. A clinical trial found that a model integrating genetics, blood biomarkers, and lifestyle data identified more suitable dietary supplements for adults classified as overweight or obese than standard physician-guided care, resulting in greater weight loss (282). The PREVENTOMICS system uses metabolomics and genetics data to classify individuals into metabolic types and assign diets tailored to these profiles; however, early trials found similar short-term weight loss in individuals assigned to a healthy control diet (283). Although these approaches reduce reliance on self-reported intake and subjective clinical judgment, they may still be influenced by biomarker variability and metabolic subtype misclassification. The inconsistent findings suggest that biomarker-based personalization alone is not sufficient if models fail to account for measurement error and uncertainty. Incorporating repeated or calibrated measurements and explicitly modeling uncertainty may improve prediction reliability and increase the likelihood of sustained weight-loss benefits.

### 7.5 Example 5: cardiometabolic risk reduction

In an attempt to address the substantial inter-individual variability in cardiometabolic responses to identical foods, researchers have developed AI-based nutrition models intended to improve cardiometabolic risk markers, including triglycerides, low-density lipoprotein (LDL) cholesterol, blood pressure, and inflammatory biomarkers (29). A randomized controlled trial compared conventional nutritional guidelines with personalized dietary recommendations based on measured cardiometabolic responses to a test meal, microbiome data, and health history. Individuals who received personalized guidance experienced greater reductions in triglycerides, body weight, and waist circumference than those who received standard guidance, although changes in LDL cholesterol were comparable between groups (284). Collectively, these results suggest that AI-driven strategies tailored to an individual's cardiometabolic response profile may yield modest but clinically meaningful improvements in risk markers. However, response-based personalization depends on the accuracy and stability of the underlying biomarkers and test-meal response measures, which may be affected by biological variability and measurement error. Incorporating repeated or standardized measurements and explicitly modeling uncertainty in response estimates may improve both prediction reliability and cardiometabolic outcomes.

### 7.6 Example 6: machine learning recalibration of wearable energy expenditure

To address systematic EE error, O'Driscoll et al. (285) applied ML-based recalibration to consumer wearable signals benchmarked against indirect calorimetry. Random forest models integrating raw accelerometry and physiological inputs substantially outperformed device-provided estimates. For the SenseWear Armband, median absolute percentage error decreased from 33.6% to 18%–19%, corresponding to a 40%–45% relative error reduction, with correlations of *r* ≥ 0.85 and root mean squared errors of 1.0–1.37 metabolic equivalents across activities. The ML model offers explicit measurement-error correction by learning and removing systematic, device- and activity-specific bias. Tekwe et al. (212), using the same SenseWear Armband in a different population and setting, offer complementary evidence demonstrating that ignoring measurement error attenuates functional regression coefficients relating EE to BMI (212). Compared with naïve estimates, the measurement error–corrected models produced coefficient functions that were meaningfully larger in magnitude across substantial portions of the activity window and exhibited distinct temporal patterns. Furthermore, the corrected effects continued to be detectable during time intervals for which naïve estimates attenuated toward zero. Simulation studies showed that uncorrected models exhibited increased bias and diminished ability to detect true exposure–outcome relationships with larger measurement error magnitudes, whereas models using IV-based correction yielded markedly lower bias, improved coverage, and more stably recovered underlying EE effects. These results provide convergent empirical and methodological evidence that correcting for measurement error in SenseWear-derived EE data, whether through ML recalibration or statistical measurement-error modeling, can substantially improve EE estimation, a critical component of energy-balance models. AI-based precision nutrition models are encouraged to either recalibrate wearable EE data using ML or explicitly model measurement error to avoid attenuating diet–activity associations.

### 7.7 Example 7: deep learning correction of wrist-worn accelerometry in free-living adults

Wrist placement introduces intensity-dependent error when using traditional regression models. Nawaratne et al. (286) developed a convolutional neural network (CNN) trained on raw triaxial wrist accelerometer data to predict EE and physical activity intensity in free-living adults, achieving a higher correlation (*r* = 0.86) for EE prediction than a standard regression model (*r* = 0.71) and improving intensity classification accuracy by approximately 10%–15%. Weekly CNN-derived estimates of moderate-to-vigorous physical activity (MVPA) were not statistically different from those derived using the reference method, whereas regression models showed systematic bias, suggesting that the CNN reduced misclassification errors that became additive over time in the simpler regression model. Although not framed as a formal measurement error–correction model, the CNN approach implicitly corrected for measurement error by learning nonlinear mappings that denoise wrist-specific artifacts. End-to-end DL models trained against reference standards can function as an implicit measurement error–correction layer for complex or non-classical error structures.

### 7.8 Example 8: machine learning and measurement-error correction of accelerometer misclassification among young children

In preschool-aged children, cut-point methods, which classify activity intensity using fixed thresholds of accelerometer counts derived from laboratory studies, often misclassify intermittent movements. Ahmadi and Trost (287) compared random forest classification models with eight cut-point methods using video-coded movement measures based on the Children's Activity Rating Scale. ML models achieved weighted kappa statistics of 0.76 (hip) and 0.72 (wrist), indicating substantial agreement with observed activity intensity, whereas cut-points had kappa statistics of 0.38–0.49 (hip) and 0.31–0.44 (wrist). The ML model also achieved 83%–88% accuracy in classifying sedentary and light-intensity activity and 68%–78% accuracy for classifying MVPA, improving classification accuracy by 20%–40% over cut-point classification. When estimating time spent engaging in activity by intensity (e.g., sedentary, light activity, MVPA), ML-based estimates were statistically similar to directly observed time, whereas estimates using the cut-point method showed systematic bias. Although the ML model addressed classification-based measurement error, rather than correcting for continuous EE bias, this finding demonstrates that misclassification error can propagate into downstream energy-balance modeling. Therefore, precision nutrition models are encouraged to avoid cut-point–derived activity inputs in populations with high movement variability.

## 8 Equity and responsible AI in precision nutrition

Personalized, precision nutrition has the potential to transform dietary guidance. However, if AI systems fail to account for bias or are trained on incomplete or unrepresentative data, even the most technically sophisticated systems can perpetuate harm and exacerbate health disparities for marginalized populations who already experience disproportionate burdens of diet-related chronic disease. Therefore, equity and responsible AI are foundational imperatives for the future of precision nutrition. Addressing equity in AI-enabled nutrition systems requires a multidimensional approach that integrates data diversity, algorithmic fairness, transparent communication, and participatory design. By embedding principles of fairness, transparency, inclusivity, and accountability at every stage throughout the AI lifecycle, we can develop precision nutrition tools that are effective, equitable, ethical, and trusted by all communities they aim to serve. To promote equity, we recommend [1] diversifying reference databases, [2] correcting for batch and platform effects, [3] stratifying model evaluation by demographics, and [4] incorporating measurement uncertainty into predictive models.

### 8.1 Conceptualizing equity across multiple levels

Evaluating equity in analytic approaches requires acknowledging that numerical balance is not equivalent to fairness, validity, or equity. For example, Obermeyer et al. demonstrated that a widely used population health algorithm exhibited substantial racial bias because it used healthcare cost as a proxy for health need; however, remedying that disparity would have increased the percentage of Black patients identified for additional care from 17.7% to 46.5% (55). In some contexts, sex- or gender-related differences may be causally relevant to the biological or behavioral process under study. In these contexts, enforcing parity or applying gender-blind analytic approaches may ignore relevant heterogeneity and inadvertently reinforce inequities, reducing model validity and fairness, especially when data quality, measurement error, or model performance differ across groups. Gender-sensitive approaches explicitly assess whether data quality, model calibration, or recommendations differ across groups and whether differences reflect broader structural conditions, whereas gender-specific approaches can address differences in biology, social structures, or care contexts that might materially affect prediction, interpretation, or intervention design. How gender equality is defined and measured can substantially influence which forms of inequity are recognized, reinforcing the need for conceptual precision in equity-oriented model design and evaluation (288).

However, gender alone is insufficient to capture the full range of equity concerns relevant to AI-enabled precision nutrition. Consistent with socioecological approaches to health equity, which emphasize that digital health systems are shaped by multiple, nested levels of influence rather than by individual-level data alone (289), equity in AI-enabled precision nutrition must be addressed at macro, meso, and micro levels that extend beyond subgroup representation. Macro-level inequities can be introduced by structural differences in policies, reimbursements, regulations, data governance, or access to high-quality devices and care. Meso-level inequities may emerge in response to differences in institutional workflows, health system implementations, reference dataset selection, or device ecosystems. Micro-level inequities may reflect differences in lived experiences, trust, digital literacy, usability, or user–model interactions. A dataset that appears balanced across sex or gender may still embed structural imbalances in age, ancestry, socioeconomic position, care access, disease severity, device quality, or measurement reliability. Intersecting dimensions, such as race, ethnicity, age, socioeconomic position, geography, language, and access to care, must also be considered, and fairness assessments should not rely solely on broad binary groupings that may obscure important within-group heterogeneity. Therefore, equitable AI evaluation should focus on calibration, validity, and subgroup performance rather than subgroup counts alone.

Established healthcare quality models, such as Donabedian's structure–process–outcomes framework (290), can be applied to AI-enabled precision nutrition, with structure encompassing the composition of training and reference datasets, device ecosystems, validation infrastructures, and access to high-quality digital tools; process comprising calibration, batch correction, uncertainty quantification, subgroup auditing, fairness-aware model development, and participatory design; and outcomes including predictive validity, subgroup fairness, user trust, clinical usefulness, and downstream behavioral or health effects. Positioning AI-enabled precision nutrition within this framework clarifies that equity requires evaluation of both the model outputs and the systems, workflows, and data environments that contribute to these outputs (290).

### 8.2 Disproportionate impact of measurement error

Measurement error frequently mirrors and magnifies structural inequities and systematic biases in how health behaviors and biological signals are captured, interpreted, and modeled, disproportionately affecting populations underrepresented in science. For example, due to variability in light absorption, the photoplethysmography-based sensors used in most commercial wearables are less accurate in individuals with darker skin tones, resulting in underestimation of heart rate and EE and affecting downstream predictions (46, 291). The underreporting of energy intake is more prevalent among individuals with higher BMI, and recall bias varies by race, educational attainment, and socioeconomic status (44, 292), introducing systematic bias into the dietary datasets commonly used to train AI models. Similarly, systematic bias exists within genomics and microbiome reference datasets, which remain heavily skewed toward individuals of European ancestry, limiting model transferability and reducing prediction accuracy among non-European populations (50, 51). These disparities contribute to unequal model performance, compromise the reliability of AI-generated recommendations in diverse populations, and reinforce structural barriers to health equity.

### 8.3 Principles of responsible AI in nutrition science

Responsible AI design in nutrition aligns with international frameworks from the WHO, AMA, and NIH, which emphasize ethical, equitable, and transparent deployment of health technologies, focusing on the following key principles.

1. Fairness and non-discrimination, requiring AI systems to be evaluated and validated for performance across demographic subgroups, using tools such as demographic parity, equalized odds, and subgroup calibration, to ensure that models do not exacerbate health disparities.
2. Transparency and explainability, requiring that models support user agency and informed decision-making by producing interpretable outputs, particularly when deployed in behavioral or clinical contexts, through the inclusion of explanatory notes or indicators of prediction confidence.
3. Accountability and oversight, requiring that developers maintain rigorous documentation of data sources, modeling decisions, and updating protocols to ensure traceability and reproducibility, which can be evaluated through regulatory review and support long-term public trust.
4. Inclusivity and co-design, in which development teams engage diverse stakeholders, including community leaders, behavioral scientists, dietitians, and end-users, to ensure that digital nutrition platforms reflect lived experiences and cultural values.

### 8.4 Open science and transparent research practices

Responsible AI in precision nutrition also requires adherence to open science principles, which reinforce the goals of responsible AI by promoting transparency, reproducibility, equity, and public trust, ensuring that precision nutrition systems remain accountable, scientifically grounded, and broadly beneficial. Open science frameworks emphasize that the validity and fairness of AI systems depend on both the model architecture and the sharing, documentation, and governance of data and methods (94, 293–295). These principles are increasingly prioritized by global scientific bodies, including NIH, WHO, and the European Commission, especially for digital health and AI research (59, 296–298). Key components include.

1. Findable, Accessible, Interoperable, Reusable (FAIR) Data Standards, which provide rigorous guidelines for structuring and sharing multimodal datasets, including dietary images, wearable signals, biomarkers, and omics data, to facilitate independent verification and reuse by other researchers (293, 294). FAIR-compliant metadata also enhances the identification of measurement-error sources across cohorts and platforms.
2. Transparent methods and the use of public code repositories to share both measurement error–correction methods and detailed methodological documentation enable reproducibility, facilitate independent calibration analyses, and support regulatory evaluation (295, 299).
3. Model documentation and responsible reporting, using tools aligned with reporting guidelines [e.g., TRIPOD-AI and CONSORT-AI (270)], such as Model Cards (300), Data Sheets (301), and nutrition-specific calibration reports, strengthen transparency by describing data provenance, preprocessing decisions, known biases (e.g., skin-tone–related wearable inaccuracies), and subgroup performance.
4. Open benchmarking using public datasets, particularly those that include culturally diverse foods, a range of skin tones, multiple wearable brands, and heterogeneous microbiome profiles, enables fair comparison across algorithms and supports rigorous auditing of subgroup-specific performance (94, 95), which are essential for identifying demographic-specific measurement-error patterns.
5. Community-engaged and participatory data governance models that support developing datasets with communities, particularly when research involves populations that are historically underrepresented in science, support the incorporation of cultural contexts into dietary databases, enhancing trust and reducing extractive data practices (96, 298).

### 8.5 Strategies for equitable AI implementation

A comprehensive strategy for equitable AI in precision nutrition must include intervention at the data, algorithmic, design, and community levels. Key goals will include the following:

1. Data diversification and subgroup validation will be important to ensure the generalizability of any developed models. To ensure generalizability, data inputs are encouraged to reflect the racial, ethnic, geographic, and socioeconomic diversity of all populations intended to benefit from AI-based precision nutrition models. Subgroup performance audits are critical for identifying any areas in which models may underperform and potentially cause harm to specific subgroups. Tools, such as FairLens (302), operationalize audits by stratifying models by attributes, such as ethnicity, sex, and insurance, demonstrating how subgroup analysis can identify and explain errors before deployment.
2. Demographic-specific calibration, using calibration equations stratified by race, sex, BMI, or other factors associated with heightened risk of measurement error, can be used to adjust wearable or dietary data. Correction methods have been developed and can be applied to mitigate batch effects and enhance comparability of high-throughput omics data across labs or cohorts (27).
3. Fairness-aware modeling techniques that employ strategies such as reweighting, adversarial debiasing, and fairness-constrained loss functions can reduce model bias at the training stage (303), which can minimize performance gaps while preserving predictive accuracy across groups.
4. The development of accessible and inclusive interfaces is recommended to accommodate a range of health literacy levels, language backgrounds, and digital proficiencies. Participatory design approaches can help co-create systems that are intuitive, respectful of user preferences, and contextually relevant.
5. Prioritizing community engagement, particularly in communities that are historically underrepresented in biomedical research, will encourage participation in all stages of development, aligning system priorities with lived realities. Community-based participatory research models offer a blueprint for integrating local expertise, fostering trust, and enhancing adoption (304).

### 8.6 Policy and research recommendations

To advance equity and responsible innovation, policy and research ecosystems must reinforce these principles.

1. Funding agencies are encouraged to require data equity plans, incentivize subgroup-focused analyses, and reward methodological transparency during grant review.
2. Regulators, such as the FDA and EMA, are encouraged to mandate demographic performance reporting and conduct equity impact assessments when reviewing and approving AI-based digital nutrition tools.
3. Journals are encouraged to enforce transparent reporting guidelines, such as extensions of CONSORT-AI or TRIPOD-AI, that are tailored for nutrition research, in addition to prioritizing equity-focused methodological work.
4. Researchers are encouraged to investigate how measurement error propagates differently across populations, assess the long-term behavioral impacts of algorithmic nutrition tools, and explore safeguards to ensure responsible model deployment at scale.

## 9 Limitation of this review

A limitation of this review is its narrative and conceptual nature. Although this review is intended to synthesize interdisciplinary evidence across nutrition science, digital health, measurement error, and algorithmic fairness to develop a practical framework for AI-enabled precision nutrition, this review was not designed as a formal, systematic review. As such, it does not rely on a prespecified search strategy or formal study-selection protocol. In addition, this review was not intended to provide a comprehensive evaluation of all dietary exposures, nutrition-related risk factors (e.g., ultra-processed food consumption), or causal relationships between precision nutrition and specific human health outcomes. Because the primary purpose of this review is to clarify how measurement error across dietary, wearable, biomarker, and multiomics data can affect the performance, fairness, and interpretation of AI-enabled precision nutrition systems (40–42, 54, 55), other topics, such as the comparative and cost effectiveness of different interventions and specific dietary controversies, are discussed only insofar as they intersect with the manuscript's methodological focus. In addition, research and literature surrounding AI-based digital health tools, regulatory guidance, and newer multimodal systems are evolving rapidly, which may limit the completeness of this review and may lead to some examples or recommendations becoming outdated over time. Nevertheless, we believe this approach is appropriate for clarifying conceptual links across fields that are often studied separately (58, 59).

## 10 Conclusion

Recent advances in wearable sensors, multiomics technologies, and ML are driving the transformative potential of scalable, adaptable, AI-driven precision nutrition models. These models are seek to optimize downstream health outcomes by generating personalized nutrition recommendations based on the real-time integration of complex data spanning biology, behavior, environment, and lived context across clinical, research, and consumer settings. However, the success, fairness, and overall impacts of these tools depend on the quality and integrity of the underlying data. Persistent measurement inaccuracies in the underlying datasets can compromise accuracy, hindering both individual personalization and broader population-level analyses.

To date, insufficient evidence has been generated to provide a comprehensive understanding of how error-corrected, unbiased, AI-enabled precision nutrition impacts health outcomes across diverse real-world settings, particularly in comparison with other established public health approaches (6, 58). Therefore, future work should pair methodological advances with longitudinal validation, subgroup-specific evaluation, and comparative effectiveness research to determine how and when AI-enabled precision nutrition systems can offer meaningful added value beyond the benefits achievable through established, lower-cost behavioral and community-based interventions (59, 270, 302).

This review synthesizes key sources of measurement error across various data types and highlights how efforts to measure and address measurement error have resulted in a growing toolbox of statistical and ML correction approaches, including RC, IV, Bayesian hierarchical models, DAEs, MTL, and hybrid models. In addition, this review highlights how measurement error can propagate through AI systems if left uncorrected. We propose a unified conceptual framework for AI systems that considers, measures, and corrects for measurement error across multiple stages of data collection, analysis, and integration. Building on and complementing existing frameworks surrounding data equity, such as the FAIR data principles (305), which emphasize mechanisms for ensuring data accessibility and interoperability, our framework focuses on how data quality can impact model fairness, interpretability, and reliability. Our work also complements emerging approaches for promoting equity and responsible innovation, such as federated learning and human-centered AI frameworks [e.g., HiCARE (306)]. Federated learning approaches attempt to enhance equity and privacy by enabling model training across decentralized data sources; however, these approaches do not address systematic differences in measurement quality across devices, populations, or environments. Human-centered AI frameworks, such as HiCARE, emphasize responsible AI design, user interaction, and systems-level integration, but these frameworks operate on the assumption of reliable and accurate input data (306). Our framework extends these approaches by first acknowledging the risk that uncorrected error poses to fairness, equity, and accurate interpretation and then explicitly modeling how measurement error originates and propagates across multimodal data pipelines. Our framework positions measurement error correction as a prerequisite for achieving fairness, interpretability, and robustness in AI-enabled precision nutrition systems.

Ensuring equitable and trustworthy AI systems requires transparent model development, inclusive design, and rigorous validation across diverse populations. More generally, removing imbalance is not equivalent to removing bias, and fairness interventions must be context-sensitive rather than defined by subgroup parity alone. Healthcare algorithms that appear neutral can still encode major disparities when they rely on biased proxy variables, as illustrated by evidence that the use of healthcare costs as a proxy for illness burden in a widely used population health algorithm results in the systematic underestimation of the needs of Black patients (55).

Moving forward, researchers, developers, regulators, and funders must collaborate to integrate measurement-error correction, demographic representativeness, and uncertainty-aware modeling throughout the entire lifecycle of precision nutrition systems. Ultimately, the success of AI in precision nutrition will be defined not by sophisticated algorithms but by scientific robustness, ethical standards, and a dedication to health equity. Recognizing and addressing measurement error will be essential for establishing credible, trustworthy, and inclusive nutrition platforms capable of realizing personalized health benefits for all.
